# Endometrial Proliferative Phase-Centered View of Transcriptome Dynamics across the Menstrual Cycle

**DOI:** 10.3390/ijms25105320

**Published:** 2024-05-13

**Authors:** Apostol Apostolov, Mladen Naydenov, Aive Kalinina, Maria Nikolova, Merli Saare, Elina Aleksejeva, Nadezhda Milova, Antoan Milov, Andres Salumets, Vesselin Baev, Galina Yahubyan

**Affiliations:** 1 Competence Centre on Health Technologies, 50411 Tartu, Estonia; apostol.apostolov@ccht.ee (A.A.); merli.saare@ut.ee (M.S.); elina.aleksejeva@ccht.ee (E.A.); andres.salumets@ccht.ee (A.S.); 2 Division of Obstetrics and Gynecology, Department of Clinical Science, Intervention and Technology (CLINTEC), Karolinska Institute, 17177 Stockholm, Sweden; 3 Department of Gynecology and Reproductive Medicine, Karolinska University Hospital, 17165 Stockholm, Sweden; 4 Department of Human Anatomy and Physiology, Faculty of Biology, University of Plovdiv, 4000 Plovdiv, Bulgaria; mnaydenov@uni-plovdiv.bg; 5 South Estonia Hospital, 65526 Võru, Estonia; kalininaaive@gmail.com; 6 Center for Women’s Health, 4000 Plovdiv, Bulgaria; maria.nikolova@cwh-bg.com (M.N.); nadezhda.milova@cwh-bg.com (N.M.); antoan.milov@cwh-bg.com (A.M.); 7 Department of Molecular Biology, Faculty of Biology, University of Plovdiv, 4000 Plovdiv, Bulgaria; baev@uni-plovdiv.bg; 8 Department of Obstetrics and Gynecology, Institute of Clinical Medicine, University of Tartu, 50406 Tartu, Estonia

**Keywords:** endometrial cycle, mid-proliferative phase, late proliferative phase, early secretory phase, mid-secretory phase, late secretory phase, transcriptome profiles

## Abstract

The endometrium, the inner mucosal lining of the uterus, undergoes complex molecular and cellular changes across the menstrual cycle in preparation for embryo implantation. Transcriptome-wide analyses have mainly been utilized to study endometrial receptivity, the prerequisite for successful implantation, with most studies, so far, comparing the endometrial transcriptomes between (i) secretory and proliferative endometrium or (ii) mid-secretory and early secretory endometrium. In the current study, we provide a complete transcriptome description of the endometrium across the entire menstrual cycle and, for the first time, comprehensively characterize the proliferative phase of the endometrium. Our temporal transcriptome analysis includes five time points including the mid-proliferative, late proliferative (peri-ovulatory phase), early secretory, mid-secretory, and late secretory phases. Thus, we unveil exhaustively the transitions between the consecutive proliferative and secretory phases, highlighting their unique gene expression profiles and possible distinct biological functions. The transcriptome analysis reveals many differentially expressed genes (DEGs) across the menstrual cycle, most of which are phase-specific. As an example of coordinated gene activity, the expression profile of histone-encoding genes within the HIST cluster on chromosome 6 shows an increase in cluster activity during the late proliferative and a decline during the mid-secretory phase. Moreover, numerous DEGs are shared among all phases. In conclusion, in the current study, we delineate the endometrial proliferative phase-centered view of transcriptome dynamics across the menstrual cycle. Our data analysis highlights significant transcriptomic and functional changes occurring during the late proliferative phase—an essential transition point from the proliferative phase to the secretory phase. Future studies should explore how the biology of the late proliferative phase endometrium impacts the achievement of mid-secretory endometrial receptivity or contributes to molecular aberrations leading to embryo implantation failure.

## 1. Introduction

The endometrium, characterized by its self-renewing nature, undergoes cyclical histological and functional transformations in growth and differentiation to prepare for embryo implantation. The endometrial cycle aligns with follicular maturation and oocyte ovulation, with the uterine and ovarian phases mirroring each other. These phases include the menstrual phase, the proliferative phase (both combined as follicular phase), and the secretory phase (luteal phase), further divided into early, mid-, and late secretory phases based on histological and molecular assessments [1].

Endometrial receptivity (ER) occurs within a brief window, influenced by estradiol and progesterone, during which the endometrial tissue becomes favorable to embryo implantation [2]. For decades, efforts in genome-wide expression studies have focused on deciphering the endometrial transcriptomic signature associated with receptivity. Despite thorough consideration of various clinical and laboratory factors, so far, unidentified genomic factors may hinder achieving a natural or medically assisted pregnancy, uncovering how the chance for conception can be enhanced.

Based on the accumulated knowledge over the past two decades, it has become increasingly evident that the cellular features of the endometrial cycle correlate with the distinct transcriptional profiles of the entire tissue, which is essential for optimal endometrial function. While several research groups have conducted detailed studies of endometrial molecular dynamics in the natural cycle, the consensus on the gene expression profile indicative of ER remains still elusive [3]. The absence of consensus could stem from experimental discrepancies, such as variations in biopsy collection and timing, tissue cellular heterogeneity, methods employed for measuring gene expression, and statistical approaches used in data analysis and interpretation, among other factors [3,4,5]. For a long time, transcriptomic studies aimed at characterizing the healthy endometrium at the genomic level used microarray-based technologies [6,7,8,9,10]. Nowadays, these studies rely on massively parallel shotgun RNA sequencing, which allows for more comprehensive analysis with increased depth and specificity [11,12]. Recent advancements in single-cell-resolution transcriptomics have also allowed for the identification of distinct cell populations within the endometrium based on their distinct transcriptomes [13,14]

A substantial proportion of the past genomic research focuses on the window of implantation (WOI) when the endometrium is functionally competent to receive the embryo, by comparing the endometrial genome expression profiles during the two critical phases including (i) the mid-secretory versus early secretory endometrium or (ii) the secretory versus proliferative phase endometria [3,5,15]. Still, the proliferative phase, which lasts for two weeks, is much less studied and is traditionally simplified as continuous tissue growth in response to estradiol stimulation rather than a complex tissue transformation into the secretory phase endometrium. In contrast to the secretory phase, fewer studies are available on the time-critical genomic factors determining the transformation of proliferative phase endometrial tissue [16]. Moreover, the proliferative endometrium also includes the peri-ovulatory time period, when sperm cells transiting the uterus aim to approach the ampulla region of the fallopian tube where oocyte fertilization is believed to take place. The supportive role of the endometrium in sperm passage before fertilization has not been studied much but deserves future attention.

Moreover, it has been suggested that the transcriptomic signature of the proliferative phase endometrium in the controlled ovarian hyperstimulation cycles in IVF may predict the pregnancy outcome following fresh embryo transfer [17]. Additionally, the aberrations found in transcriptomic profiles at the WOI in patients with recurrent implantation failure in IVF indicate decreased cellular proliferation, a phenomenon typically observed in the proliferative phase endometrium [18]. This emphasizes the crucial need to elucidate the dynamics of endometrial gene expression throughout the menstrual cycle, with a particular focus on studies involving proliferative phase endometrial tissue. Such efforts would substantially enhance our comprehension of achieving endometrial receptivity during the subsequent secretory phase of the uterine cycle.

Data on transcriptional profiles across various time points of the proliferative and secretory phases within a single study utilizing the same analytical method are scarce in the literature. In this study, we performed a comprehensive transcriptome analysis of whole-tissue endometrium across the menstrual cycle using RNA exome sequencing and put specific effort into involving more than a single biopsy from the proliferative phase of the uterine cycle. Therefore, the analysis covered five time points of the endometrial cycle, covering the mid-proliferative (MP), late proliferative (LP) or peri-ovulatory time period, early secretory (ES), mid-secretory (MS), and late secretory (LS) phases. The analysis involved identifying differentially expressed genes (DEGs) at each time point by comparing them pairwise with the mid-proliferative (MP) phase. The endometrium undergoes dynamic changes from the MP phase to the LP phase, preparing for the potential implantation of a fertilized egg. Utilizing MP as a reference allowed us to perform comparative transcriptomic profiling, concentrating on the alterations occurring during the proliferative phase and gaining insights into its significance for the subsequent maturation of the endometrium. DEGs were either specific to a particular phase or shared across all time points. Furthermore, the chromosomal locations of these DEGs were examined to detect co-expressed clusters of genes. Understanding these transcriptomic patterns is fundamental within the tissue. Gene Ontology and hallmark gene enrichment analysis were conducted on DEGs to uncover the functional alterations occurring throughout the endometrial cycle.

## 2. Results

### 2.1. The Transcriptional Landscape of the Endometrial Cycle Unveiled Distinct Changes during the LP and MS Phases

**Figure 1:**
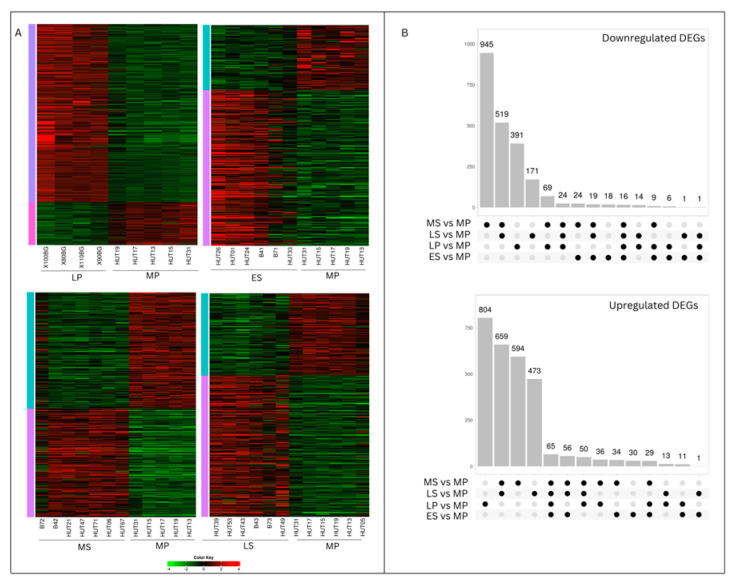
Time-dependent alterations in gene expression within the healthy endometrium across the menstrual cycle. (**A**) Heatmap of log2FC values of differentially expressed genes (DEGs) in the late -proliferative (LP), early-secretory (ES), mid-secretory (MS), and late-secretory (LS) phases compared to the mid-proliferative (MP) phase; (**B**) UpSet plot of DEGs across endometrial cycle phases. Intersection of sets of genes at endometrial cycle phases. Each column corresponds to a time point (one dot) or set of time points containing the same DEGs.

We employed RNA-exome sequencing to examine how gene expression levels vary across different menstrual cycle phases in healthy human endometrium. We identified 5082 genes that were significantly differentially expressed (DEGs) between the control group (MP phase) and the LP, ES, MS, and LS phases (Supplemental Table S1, Table 1). To visualize these expression changes in each phase, we created heat maps focusing on the statistically significant DEGs (*p*adj < 0.05) (Figure 1A).

Phase-specific and shared DEGs were identified and visualized using an Upset Diagram (Figure 1B). Notably, many significant DEGs were uniquely associated with two time points—the LP and MS phases. The highest number of phase-specific DEGs was observed in the MS phase, with downregulated genes (945) outnumbering upregulated genes (594). Another notable time point with many specific DEGs was the LP phase, where upregulated genes (804) exceeded downregulated genes (391), unlike the MS phase. The most shared DEGs (1178) were found between the MS and LS phases. Additionally, a set of 81 genes exhibited consistent differential expression throughout the entire endometrial cycle.

**Figure 2:**
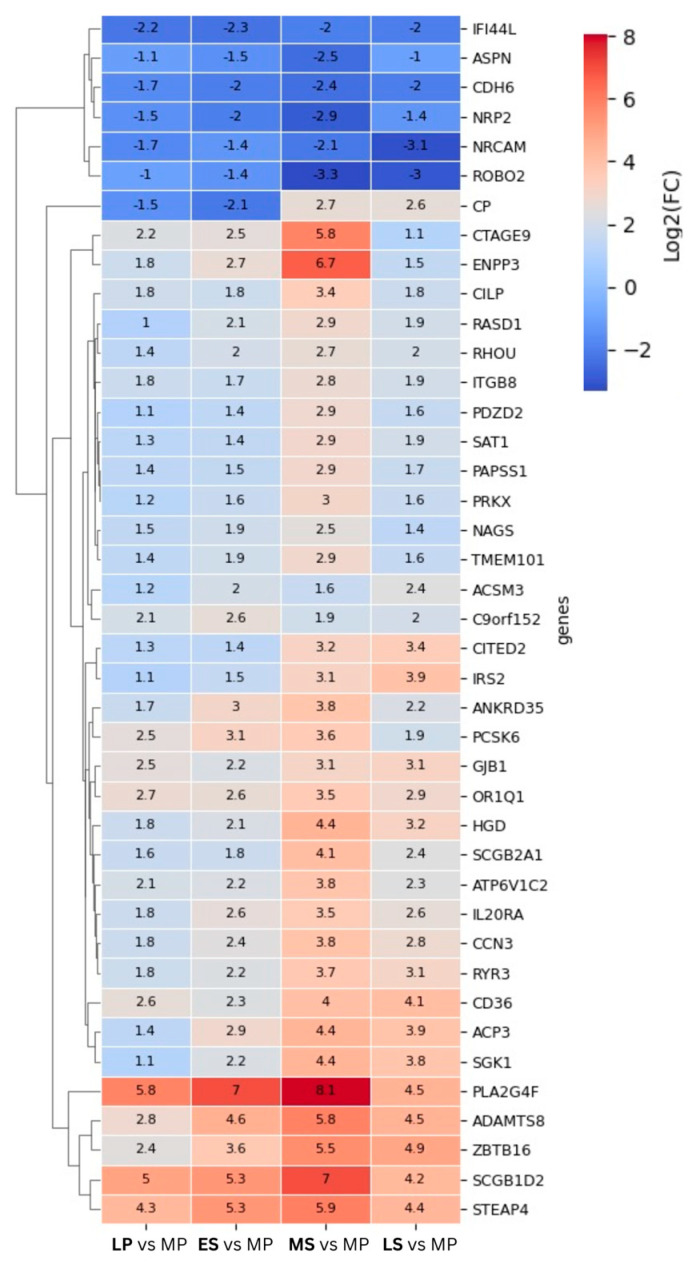
Heatmap of log2FC values of the top differentially expressed genes (DEGs) shared among the phases of the endometrial cycle. The late-proliferative (LP), early-secretory (ES), mid-secretory (MS), and late-secretory (LS) phases are compared to the mid-proliferative (MP) phase; log2FC ≥ 2| in at least one time point across the dataset of four time points; *p*adj < 0.05.

To illustrate the fluctuating response of the DEG throughout the endometrial cycle, a log2FC heat map of DEGs shared across all time points was created (Figure 2). Before clustering, we refined the selection of genes of interest, ensuring inclusion criteria that required a log2FC≥ |2| in at least one time point across the dataset of four time points, resulting in 42 DEGs.

Most of the shared DEGs demonstrate elevated expression levels throughout the menstrual cycle compared with the proliferative phase, indicating a prevailing trend in positive gene regulation. These genes exhibit increased expression during the LP phase, gradually rising until peaking at the WOI. Three clustering genes—*STEAP4*, *SCGB1D2*, and *PLA2G4F*—stand out among the upregulated genes. Their expression levels show a significant increase at the LP phase (with log2FC of 4.3, 5, and 5.8, resp.), reaching a maximum at the MS phase (with log2FC of 5.9 and 8.1). The transcriptional levels of a few genes remain consistently suppressed at time points following the proliferative phase, reaching their lowest expression levels at the WOI. One gene, Ceruloplasmin (*CP*), undergoes contrasting expression levels—its expression was downregulated in the LP and ES phases, then upregulated in the MS and LS phases. Another interesting observation, specific to the MS phase, was the sharp increase in the expression levels of two genes—*CTAGE9* and *ENPP3*.

To examine the expression patterns of the DEGs in more detail, we compiled a list of the top 10 upregulated and top 10 downregulated genes, as presented in Table 1. Our analysis focused on the LP and MS phases, which exhibited the most significant changes compared with the proliferative phase, regarding the magnitude of change (represented as log2) and the total number of genes with changed expression patterns. As indicated in Figure 1, the table reveals a noteworthy finding from our investigation: the top 10 upregulated and downregulated genes during the LP phase are entirely different from those in the MS phase. Remarkably, many of the top 20 genes listed in the MS phase (Table 1) align with prior findings by other researchers as DEGs [6,11,12,19].

### 2.2. Genomic Distribution of DEGs Identified Dynamic Patterns across the Endometrial Cycle

**Figure 3:**
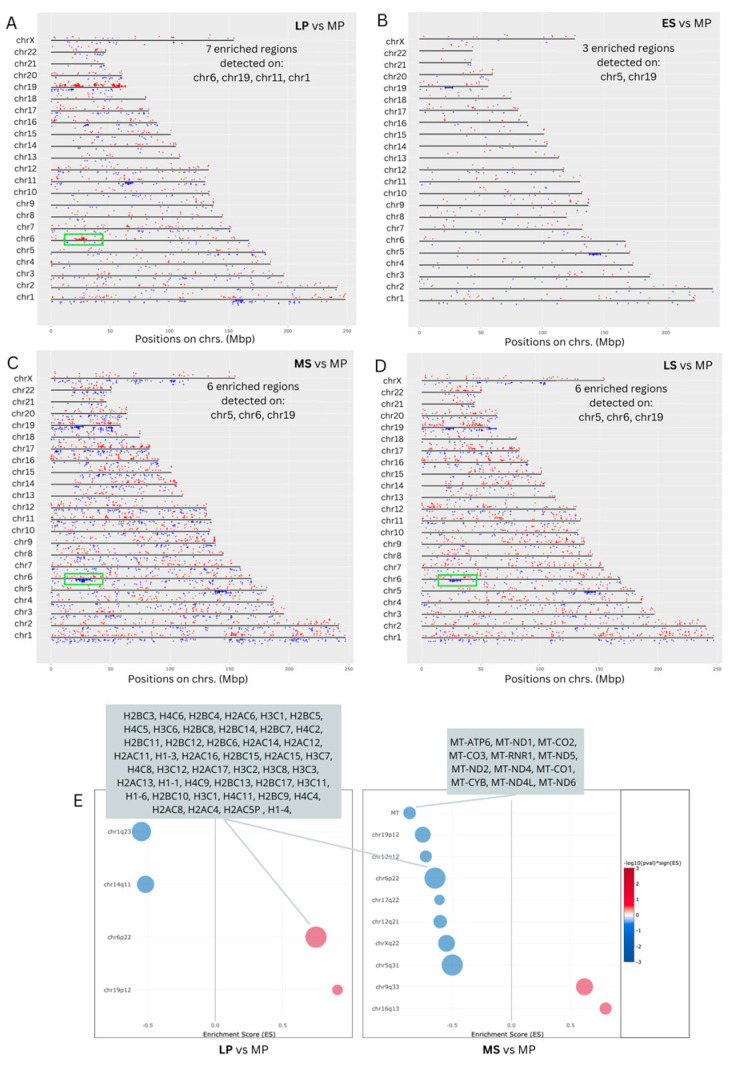
Chromosomal distribution of the differentially expressed genes (DEGs) in the healthy endometrium across the menstrual cycle. (**A**) Late-proliferative (LP) phase vs. mid-proliferative phase (MP); (**B**) early-secretory (MS) phase vs. MP phase, (**C**) mid-secretory (MS) phase vs. MP phase; and (**D**) late-secretory (LS) phase vs. MP phase. The HIST cluster is indicated in a green box. (**E**) Visualization of the chromosomal enriched regions in the LP and MS phases.

The distribution pattern of DEGs on chromosomes is depicted in Figure 3A–D. A widespread presence of DEGs across the genome was observed at all time points. The most enriched regions (seven) were observed in the LP phase on chromosomes 19, 6, 11, and 1. For the MS and LS phases, six enriched regions were found on chromosomes 19, 6, and 5. In the ES phase, where the number of DEGs was the lowest, there were three enriched regions, one on chromosome 5 and two on chromosome 19. The distinctive pattern observed in the enriched region on chromosome 6, highlighted by a green box, during the LP, WOI, and LS phases is of notable interest. Specifically, gene expression is upregulated during the LP phase and downregulated during the WOI and LS phases, offering intriguing insights. The large histone gene cluster, HIST1, on human chromosome 6 (6p21–p22) contains 55 histone genes [20], of which we identified 46 DEGs to be enriched in the cluster (Figure 3E). Furthermore, an enriched region was revealed on the mitochondrial chromosome in the MS phase, related to 12 mRNAs.

### 2.3. Functional Enrichment Exhibits Significant Alterations throughout the Endometrial Cycle

**Figure 4:**
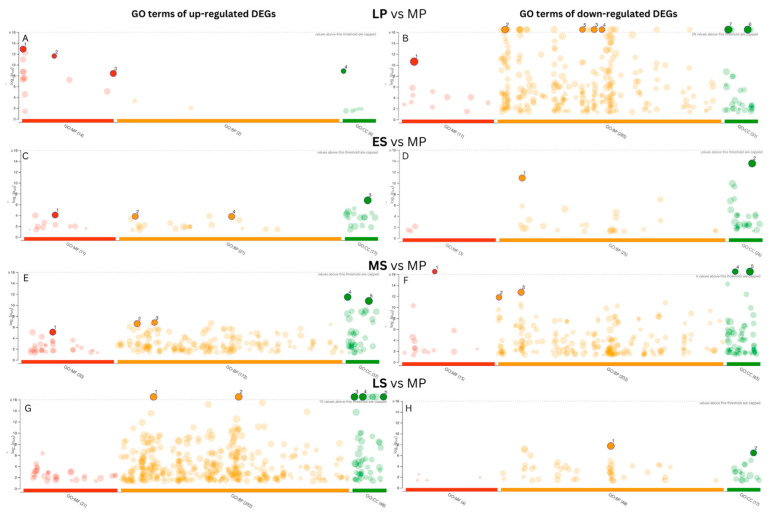
Gene Ontology (GO) classification of the differentially expressed genes (DEGs) across endometrial cycle phases. GO annotations show significant enrichment of three main categories (biological process (BP), cellular component (CC), and molecular function (MF)) with an adjusted *p*-value < 0.05. The *y*-axis indicates the number of genes in each category. (**A**) LP phase, upregulated DEGs (1—DNA-binding transcription factor activity, RNA polymerase Il-specific, 2—structural constituent of chromatin, 3—sequence-specific double-stranded DNA binding, 4—nucleosome); (**B**) LP phase, downregulated DEGs (1—protein binding, 2—immune system process, 3—leukocyte activation, 4—lymphocyte activation, 5—T cell activation, 6—cell periphery, 7—plasma membrane); (**C**) ES phase, upregulated DEGs (1—transmembrane transporter activity, 2—organic acid metabolic process, 3—cell periphery, 4—nervous system process); (**D**) ES phase, downregulated DEGs (1—cell adhesion, 2—cell periphery); (**E**) MS phase, upregulated DEGs (1—transmembrane transporter activity, 2—lipid metabolic process, 3—regulation of hormone levels, 4—extracellular space, 5—cell periphery); (**F**) MS phase, downregulated DEGs (1—cell adhesion, 2—cell periphery, 3—extracellular matrix, 4—nuclear division, 5—structural constituent of chromatin); (**G**) LS phase, upregulated DEGs (1—response to organic substance, 2—regulation of multicellular organismal process, 3—extracellular space, 4—vesicle, 5—extracellular vesicle); (**H**) LS phase, downregulated DEGs (1—system development, 2—plasma membrane region).

We conducted Gene Ontology (GO) analyses to explore the DEGs’ biological relevance and functional implications across the endometrial cycle (Figure 4). Upregulated DEGs exhibited significant enrichment in RNA polymerase II-specific DNA-binding transcription factor activity in the LP phase in the molecular process category. In contrast, downregulated DEGs showed enrichment in protein binding. Downregulated DEGs were notably enriched in immune system processes in the biological function category. Regarding the cellular component category, upregulated DEGs were associated with the nucleosome, whereas downregulated DEGs were enriched in the cell periphery.

In the ES phase, we observed that upregulated DEGs were more abundant in transmembrane transporter activity in the molecular function (MF) category, while downregulated DEGs were associated with cell adhesion and cell periphery. During the MS phase, upregulated DEGs were notably involved in transmembrane transporter activity like the ES phase. Additionally, they were associated with lipid metabolic processes, regulation of hormone levels, extracellular space, and cell periphery. Downregulated DEGs were significantly linked to cell adhesion, the extracellular matrix, nuclear division, and the structural constituent of chromatin. Moving to the LS phase, upregulated DEGs displayed more GO term enrichments than downregulated DEGs. They were mainly enriched in GO terms such as the response to organic substances and the regulation of multicellular organismal processes, as well as in extracellular space and extracellular vesicles.

**Figure 5:**
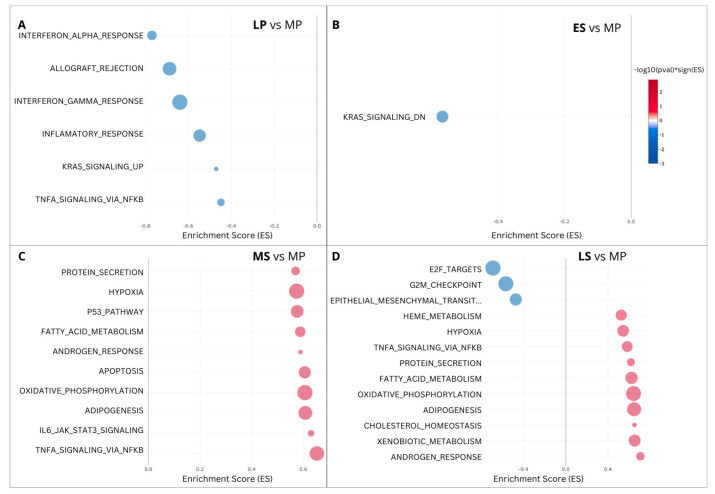
Hallmark gene enrichment of differentially expressed genes (DEGs) across the menstrual cycle. (**A**) Late-proliferative (LP) phase vs. mid-proliferative phase (MP); (**B**) early-secretory (ES) phase vs. MP phase, (**C**) mid-secretory (MS) phase vs. MP phase; and (**D**) late-secretory (LS) phase vs. MP phase.

Subsequently, we conducted a DEG set enrichment analysis, specifically targeting the Hallmark gene sets representing clearly defined biological states or processes with consistent expression patterns (Figure 5). The study unveiled a predominance of downregulated DEGs enriched in the LP phase, linked to interferon alpha and gamma response, inflammatory response, and allograft rejection. Conversely, upregulated DEG enrichment prevails in the MS phase and is associated with protein secretion, hypoxia, and oxidative phosphorylation. Notably, the TNFA signaling via NFKB exhibits a contrasting profile, downregulated in the LP phase, and upregulated in the MS phase. Furthermore, a mix of up- and downregulated gene pathways was observed in the LS phase.

## 3. Discussion

The fine-scale temporal transcriptomic analysis is a robust method for studying the genetic fluctuations that occur during the monthly cyclic changes in the human endometrium. In research, transcriptome variations are often studied by comparing the endometrial WOI phase with the early secretory or proliferative phases. The focus on the WOI is well-founded because of its critical role in preparing the endometrium for embryo implantation. Still, the endometrium, a dynamic tissue, begins preparing for the WOI and embryo implantation immediately after menstruation. Hence, it is plausible to suggest that the attainment or lack of endometrial receptivity during the mid-secretory phase is influenced by molecular processes set in motion either in the early or mid-proliferative phases, as also studied in the present study. Given the limited and fragmented data in the literature regarding transcriptomic profiles across the entire endometrial cycle, including the mid-proliferative and late proliferative (peri-ovulatory) phases, we employed high-throughput sequencing to track these alterations comprehensively across the cycle as a whole.

In the LP phase of the endometrial cycle, significant changes occur, characterized by increased cellular proliferation and differentiation, which are influenced by rising estradiol levels and maturing ovarian follicles before ovulation. These processes are influenced by rapidly increased estradiol levels from the fully matured pre-ovulatory follicle. A delicate balance exists in the endometrial tissue among proliferation, the increasing thickness of the endometrium, and tissue differentiation, initiated immediately after ovulation by the progesterone produced by the corpus luteum [21].

Our findings reveal that upregulated DEGs in the LP phase are enriched in RNA polymerase II-specific transcription factor activity and associated with the nucleosome cellular component. Specifically, the numbers of DEGs encoding histone proteins, which are part of the extensive histone gene cluster HIST1 on human chromosome 6, show elevated expression levels. Histones regulate gene expression and chromatin structure during the endometrial cycle. Studies indicate that histone acetylation, a key epigenetic modification, increases during the early proliferative (EP) phase, declines until ovulation, and then rises post-ovulation [22,23]. Here, we note fluctuations in the transcriptional profile of the HIST cluster throughout the cycle. Our research reveals a notable increase in transcripts linked to this cluster during the LP phase, contrasting with significant suppression observed during the MS phase. Among the altered transcripts, we identified the numbers of histone variants, suggesting their potential influence on replication, gene expression, and chromatin dynamics. Still, the specific roles of histone variants in endometrial transitions are unknown and require further investigation.

Moreover, during the LP phase, of the 47 DE small nucleolar RNAs (snoRNAs), 42 were increased (for example, SNORD14B, SNORA63C, and SNORA48). These snoRNAs are part of the C/D box snoRNA family, primarily modify rRNA, are involved in tRNA and mRNA modification, and influence alternative splicing [24]. Future research should also focus on elucidating the functional roles of these RNAs in achieving endometrial receptivity.

An in-depth analysis of the MS phase revealed expected changes in gene expression profiles. By meticulously examining the most significant DEGs and reviewing the relevant literature, we identified many genes previously reported by other researchers during the WOI [11,12,25]. A considerable portion of the DEGs from the MS phase displayed expression patterns like those previously established for the WOI [15]. However, some of the DEGs specific to the MS phase that we found were not documented in the existing literature. This can be due to various reasons, such as the control group used in the current study, the MP samples, against which the comparative expression analysis was performed, and the tools and settings used in bioinformatics analysis. We observed 12 mitochondrial DEGs downregulated during the MS phase, which may help modify the endometrial tissue’s energy production before embryo implantation. These changes may support the recently discovered mechanisms of vertical transmission of maternal mitochondrial DNA through extracellular vesicles from the endometrium to the embryo [26]. Moreover, a meaningful observation derived from the Hallmark enrichment analysis was the upregulation of hypoxia-related genes during the MS and LS phases of the menstrual cycle, likely indicating preparation for the upcoming tissue shedding and menstruation.

Our study unveils, for the first time, the genes exhibiting different abundancies among all five of the studied menstrual cycle phases. These genes are visualized through a heatmap illustrating their log2FC across all cycle phases (Figure 2). Most notably, many of these genes demonstrate elevated expression during the LP phase, followed by a steady increase in expression in the ES phase, culminating in peak levels during the WOI. Among these genes, *STEAP4*, *SCGB1D2*, and *PLA2G4F* exhibit notable characteristics, with their expression markedly elevated in the LP phase, followed by a further increase during the WOI. *STEAP4* (Transmembrane Epithelial Antigen of the Prostate 4) is a metalloreductase implicated in metabolism and cancer progression. Dysregulation of *STEAP4* has been linked to impaired ER in recurrent implantation failure [27] and implantation failure in cases of thin endometrial tissue [28]. *SCGB1D2* belongs to the lipophilin subfamily and is prominently expressed in organs with strong endocrine responsiveness, like the mammary glands [29]. Earlier gene expression profiling studies identified *SCGB1D2* in the endometrium, where it was noted as downregulated in the WOI and upregulated in the ES phase of the natural cycle [30]. However, our study revealed a different expression profile, likely influenced by the choice of the control groups. *PLA2G4F* is a member of the phospholipase A2 group IV family. A recent iTRAQ-based proteomics study identified PLA2G4F as a differentially expressed protein (DEP) in mid-secretory endometrium. It is categorized as one of the top five hub proteins that regulate endometrial receptivity [31]. Ceruloplasmin (CP) is the sole gene with downregulated expression in the LP phase and upregulated expression in the MS phase. CP is the primary copper-containing iron transport protein that exhibits ferroxidase activity [32], and it is noted among the upregulated genes in the MS phase [7].

The observed sharp increase in the expression level of the ENPP3 gene specifically during the MS phase aligns with previous studies where it was found to have MS-specific elevated expression in mammals [33]. This suggests it may play an important role in embryo implantation during this phase [34]. Furthermore, some authors propose this gene as a biomarker of endometrial receptivity [34] or even suggest its use in a non-invasive test of endometrial receptivity [35]. The other gene specifically highly expressed during the MS phase, *CTAGE9*, belongs to the CTAGE family (cancer/testis antigen family), which exhibits rapid and primate-specific expansion [36,37]. Many of these genes are specifically expressed in reproductive organs and germ cells and aberrantly expressed in several human cancers [38], but there are limited data in the literature regarding the expression of *CTAGE9*. One possible explanation might be that its expression is particular to the timing of endometrial processes.

Through the LP phase preceding ovulation in the menstrual cycle, immune modulation becomes essential for facilitating embryo implantation. Previously, a decrease in T cell subpopulations during the LP phase of the endometrial cycle was demonstrated, in particular, in cytotoxic T cells [16]. Here, we observed the functional enrichment of downregulated DEGs during the LP phase including processes related to the immune system such as leukocyte, lymphocyte, and T cell activation, likely reflective of decreases in T cell numbers. Conversely, in the MS phase, genes like *IL-6*, *TNFA*, and *STAT3* are upregulated.

An intriguing finding from the Hallmark enrichment analysis involves the interconnection of the NFKB, TNF, and STAT3 pathways. Notably, NFKB, activated by TNFA, interacts with STAT3, which is activated by IFNA, to enhance NFKB target gene expression [39]. These pathways, which are associated with interferon signaling, demonstrate downregulation of TNFA in the LP phase and upregulation of STAT3 and TNFα in the MS and LS phases, affirming their interconnection and potential involvement in endometrial tissue cycling. The observed downregulation of IFNs and related genes during the LP phase aligns with prior observations, indicating a transient increase in certain IFNs (e.g., IFNA) within the WOI in the human endometrium [40]. Interferons exert significant regulatory effects on cell growth, viability, and immune responses [41], with crucial roles documented in the implantation process within the normal endometrium [42].

This dynamic modulation of the immune response throughout the menstrual phases will likely contribute to significant alterations in the immune microenvironment within the endometrium. These observed changes are consistent with patterns identified by previous researchers [43,44,45,46]. This modulation may serve as a mechanism to prevent the recognition and rejection of sperm and fetal cells by maternal endometrial immune cells [43]. Such modulation is crucial for successful implantation and pregnancy maintenance [47].

## 4. Materials and Methods

### 4.1. Study Design and Participants

The study protocol received approval from the Ethics Committee of the Faculty of Biology, Plovdiv University “Paisij Hilendarski”, Plovdiv, Bulgaria, under Certificate of Approval №3/02.09.2019 and the Research Ethics Committee of the University of Tartu, Estonia (No. 330M-8). All participants provided written informed consent.

Endometrial samples were collected from 28 participants between January 2020 and September 2023. The women selected met the following specific criteria: healthy, fertile individuals aged 20 to 40 years, with regular menstrual cycles lasting 21 to 28 days and a body mass index (BMI) falling within the range of 19 to 29 kg/m^2^. They were all highly motivated volunteers without any accompanying diseases such as metabolic, endocrine, autoimmune, sexually transmitted, or gynecological infertility-associated diseases (e.g., hydrosalpinx, endometriosis, polycystic ovary syndrome, myomas, polyps, or uterine anomalies). Each participant affirmed their non-smoking status, abstention from alcohol consumption, lack of medication use, and absence of a history of febrile illness. They all had a history of normal pregnancy and at least one healthy child.

Sonographic folliculometry and endometrial thickness assessments were conducted utilizing the Fukuda Denshi Full Digital Ultrasound System UF-870AG (Tokyo, Japan), commencing on day 7 of the menstrual cycle and sequentially performed on successive days to monitor menstrual cycle progression.

### 4.2. Endometrial Biopsies and RNA Extraction

Twenty-eight endometrial biopsies were obtained from the cohort of women as follows: five biopsies from individuals in the MP phase (cd 8-10), four biopsies from individuals at the LH surge time-point (LP phase), six biopsies from participants in the ES phase (LH +2/+3), seven biopsies from subjects in the MS phase (LH +7/8, WOI), and six biopsies from women in the LS phase (LH +11/+13)) of the natural cycle. The menstrual cycle phase was confirmed by menstrual cycle history and LH peak measurements with BabyTime LH urine cassette (Pharmanova, Belgrade, Serbia). When the test yielded a positive result, the LP biopsy was performed.

Endometrial biopsy samples were obtained using a Pipelle catheter (Laboratoire CCD, Paris, France) and stored in RNAlater Solutions for RNA stabilization (ThermoFisher Sci, Waltham, MA, USA). RNA from the endometrial biopsies was extracted with a miRNeasy micro kit (Qiagen, Hilden, Germany), separating larger RNA and miRNA fractions. RNA quality and quantity were analyzed on a Bioanalyzer TapeStation 2100 with RNA ScreenTape (Agilent, Palo Alto, CA, USA). Samples with an RNA integrity number (RIN) ≥7 were considered suitable for further analysis.

### 4.3. RNA Sequencing and Bioinformatics Analysis

The whole-exome RNA library was synthesized using the TruSeq exome RNA library preparation kit from Illumina, which facilitates the enrichment of coding sequences by utilizing sequence-specific probes. For the library preparation, 100 ng of RNA was used. Once prepared, the library was pooled, and its quality was determined using Agilent’s High Sensitivity DNA ScreenTape D1000 system (Agilent, Waldbronn, Germany). Following quality assessment, we adjusted the library to a concentration of 2 nM. For the sequencing phase, 1.1 nM from the pooled library was used on the NextSeq 1000 platform, utilizing a single-end sequencing method with a read length of 80 base pairs.

Quality control of RNA sequencing data and adapter trimming for FASTQ files were performed using FastQC and Trim Galore [48]. The resulting clean reads were mapped to the GRCh38/hg38 human reference genome via HISAT2 [49]. Count matrices were generated based on genome annotation using the featureCounts tool [50] and fed into the DESeq2 package for DEG analysis. Genes showing differential expression with an adjusted *p*-value (*p*-adj) < 0.05 were selected for downstream analysis. Positional chromosome and Hallmark gene set enrichment analysis was performed using the eVITTA tool—easyGSEA (https://tau.cmmt.ubc.ca/eVITTA/easyGSEA/, accessed on 12 May 2024) [51]. The Hallmark gene set represents specific and well-defined biological processes from the Molecular Signatures Database (MSigDB) [52]. Heatmap and UpSet plots of DEGs were generated using iDEP (http://bioinformatics.sdstate.edu/idep/, accessed on 12 May 2024) [53]. GO enrichment analysis was performed using g:Profiler (https://biit.cs.ut.ee/gprofiler/gost, accessed on 12 May 2024) with the g:SCS multiple testing correction method applying a significance threshold of 0.05 [54].

## 5. Conclusions

In the current study, we provided a complete transcriptome description of the endometrium across the entire menstrual cycle and, for the first time, comprehensively covered the proliferative phase by involving both MP and LP (peri-ovulatory) samples. Thus, our study unveiled exhaustively the transitions between the consecutive proliferative and secretory cycle phases, indicating their unique gene expression profiles and possible distinct biological functions. These molecular signatures complement existing knowledge and provide new insights into the genomic determinants of the cycling endometrium. Henceforth, future research endeavors are necessary to validate the findings observed through RNA sequencing regarding genomic determinants throughout normal or dysregulated menstrual cycles. As an example of coordinated gene activity, the expression profile of histone-encoding genes within the HIST cluster on chromosome 6 showed an increase in cluster activity during the LP phase and a decline during the MS phase. In the current study, we revealed the intricate nature of the gene expression regulation in the proliferative phase. The LP (peri-ovulatory) endometrium plays a crucial role in facilitating the passage of sperm cells for oocyte fertilization. Furthermore, progesterone-driven transition commences to establish receptivity in the MS phase. Therefore, future studies should investigate the extent to which the biology of the LP phase endometrium influences the attainment of MS endometrial receptivity or contributes to molecular aberrations resulting in embryo implantation failure.

## Figures and Tables

**Table 1:** Top differentially expressed genes (DEGs) in the late proliferative (LP) and mid-secretory (MS) phases compared to the mid-proliferative phase (MP).

Gene Name	log2 FC	P adj	Study
MS vs. MP			
*ATP12A*	10.67	8.93 × 10^−11^	[12]
GLYATL3 *	9.02	1.39 × 10^−8^	[11,12]
*SULT1E1*	8.93	8.52 × 10^−14^	[12]
*PLA2G2A*	8.52	7.36 × 10^−6^	[6,11,12]
*CYP26A1*	8.42	2.01 × 10^−32^	[11,12]
*GAST*	8.27	6.37 × 10^−5^	[11,12,19]
*LRRC26*	8.15	1.46 × 10^−10^	[11]
*MT1H*	8.13	9.43 × 10^−27^	[11,12,19]
*PLA2G4F*	8.06	5.05 × 10^−30^	[11,12]
*MT1HL1*	7.88	4.04 × 10^−10^	-
*IGFN1*	−7.35	1.27 × 10^−28^	[11,12]
CDH4 *	−6.23	1.14 × 10^−7^	[11]
*CSMD3*	−6.2	1.84 × 10^−14^	[12]
*DPP10*	−6.07	1.74 × 10^−13^	[12]
LINC03010 *	−6.02	1.00 × 10^−4^	-
*GAPDHP71*	−5.95	1.02 × 10^−6^	-
*ASIC2*	−5.9	2.25 × 10^−9^	[11,12]
ECEL1P2 *	−5.85	8.07 × 10^−21^	-
BPIFB1 *	−5.83	1.87 × 10^−5^	-
SERPINB3 *	−5.83	2.66 × 10^−4^	-
LP vs. MP			
RNA5-8SN3 *	7.61	2.38 × 10^−5^	-
SNORD14B *	6.19	1.93 × 10^−12^	-
*FRG1*	6.08	3.24 × 10^−4^	-
*NLGN4Y*	6.06	2.10 × 10^−2^	-
SNORA63C *	5.80	5.43 × 10^−5^	-
*PLA2G4F*	5.80	9.48 × 10^−4^	-
*GLRXP2*	5.74	6.36 × 10^−4^	-
TRPC6P8 *	5.48	1.10 × 10^−3^	-
BRDTP1 *	5.43	2.02 × 10^−4^	-
*HMGCS2*	5.36	7.80 × 10^−4^	-
PPBP *	−5.89	5.64 × 10^−3^	-
LRRC15 *	−5.02	3.99 × 10^−5^	-
TRGJP2 *	−4.98	1.05 × 10^−2^	-
*TCL1A*	−4.73	3.28 × 10^−2^	-
*CCL22*	−4.55	6.42 × 10^−7^	-
FOSB *	−4.46	1.67 × 10^−4^	-
*KRTAP10-12*	−4.33	3.92 × 10^−2^	-
*CEACAM5*	−4.27	6.35 × 10^−3^	-
CD70 *	−4.24	4.01 × 10^−2^	-
FOS *	−4.11	4.45 × 10^−7^	-

* Phase-specific DEGs.

## Data Availability

The datasets presented in this article are not readily available because the data are part of an ongoing study.

## Supplementary Materials

The following supporting information can be downloaded at: https://www.mdpi.com/article/10.3390/ijms25105320/s1.
